# Application of Saline Infusion Sonocolpography in Diagnosis and Treatment of Perforated Transverse Vaginal Septum

**DOI:** 10.1155/2019/6738380

**Published:** 2019-07-22

**Authors:** Ayumi Ono, Iori Kisu, Tomoko Iijma, Hiroshi Senba, Kiyoko Matsuda, Motoko Katayama, Ayaka Iura, Yumiko Miura, Nobumaru Hirao

**Affiliations:** ^1^Department of Obstetrics and Gynecology, Federation of National Public Service Personnel Mutual Aid Associations, Tachikawa Hospital, Tokyo, Japan; ^2^Department of Obstetrics and Gynecology, Keio University School of Medicine, Tokyo, Japan

## Abstract

Transverse vaginal septum (TVS) is a particularly rare vaginal anomaly, and diagnosis is often difficult in a genital examination. We herein present a case of perforated TVS for which successful diagnosis and treatment were achieved using a new technique referred to as saline infusion sonocolpography. A 32-year-old female presented with primary infertility. Speculum examination revealed a blind vaginal canal with two pinpoint perforating holes. Foley catheters with inflated balloon were inserted into the two apertures, and then normal saline was injected through the catheters to distend the vaginal pouch. This procedure of saline infusion sonocolpography revealed the uterine cervix and vaginal pouch and permitted diagnosis of perforated TVS of the upper vagina. The septum was excised and a normal cervix was ascertained. The patient had no complication such as agglutination of the vagina postoperatively. This case suggests that saline infusion sonocolpography may be useful for diagnosis and treatment of TVS.

## 1. Introduction

Congenital anomalies of the genital tract arise from errors in embryogenesis and vary in anatomic features, clinical presentation, and reproductive performance. Some such anomalies are asymptomatic and may remain undetected. Transverse vaginal septum (TVS) is a rare anomaly (incidence of 1:70000) derived from failure of fusion between the vaginal palate and the caudal end of the fused Mullerian ducts [1]. Clinical manifestation of TVS depends on whether the septum is imperforated (complete septum) or perforated (incomplete septum). Imperforated TVS may be symptomatic at the early stage of life, in the neonatal period, or in puberty. Neonatally, the infant may have hydrometrocolpos due to accumulation of vaginal secretions. With onset of puberty, symptoms such as dysmenorrhea, cyclic abdominal pain, and pelvic fluid retention due to obstruction of menstruation may occur. In contrast, perforated TVS is a latent condition that is usually noticed due to an incidental finding of dysmenorrhea, dyspareunia, or infertility [2].

TVS requires differentiation from vaginal atresia/aplasia since treatments and preoperational managements for these malformations differ. However, the conventional tools such as genital examination, ultrasonography, computed tomography (CT) and magnetic resonance imaging (MRI) have the limitation as a diagnostic tool to distinguish each malformation. Hence, careful evaluation is needed before treatment, and the novel diagnostic procedure are needed to be proposed. We herein present a case of perforated TVS for which diagnosis and treatment were achieved using a new technique, saline infusion sonocolpography. Written consent from the patient was obtained for writing of this report.

## 2. Case Report

**Figure 1:**
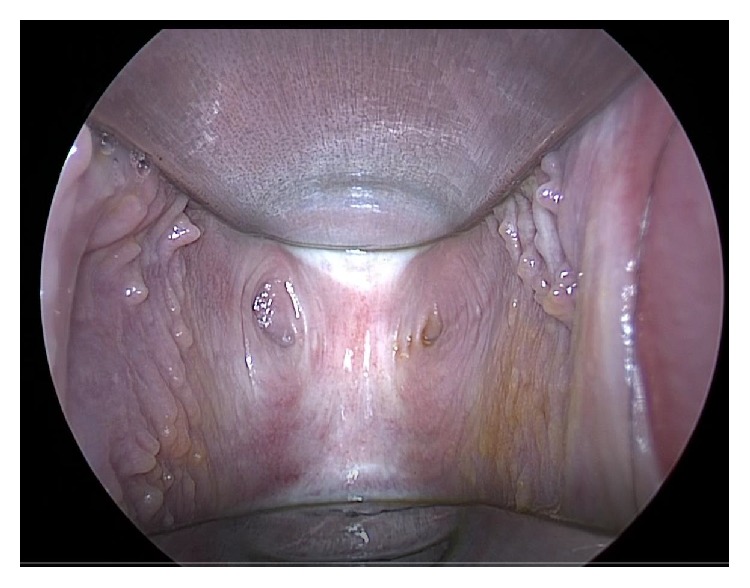
Speculum examination revealed a 7 cm blind vaginal canal of normal caliber with two bilateral 2-3 mm pinpoint perforating holes, but no visualization of the cervix.

**Figure 2:**
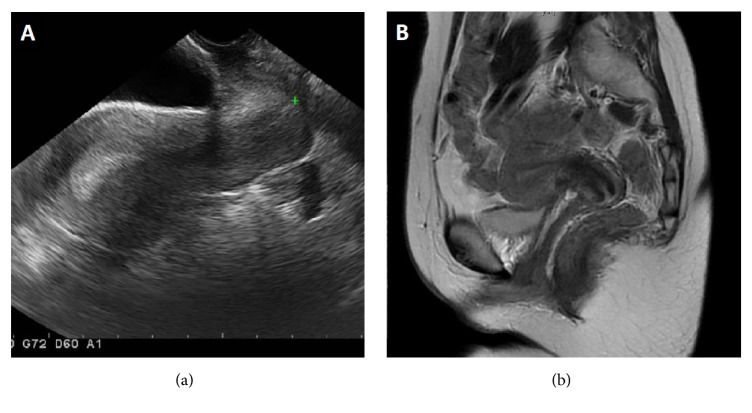
Transvaginal ultrasonography (A) and magnetic resonance imaging (B) showed a normal cervix and uterus and 2.5 cm left ovarian endometriosis, but the septum of the vagina was not detected.

A 32-year-old female, gravida 0, presented to our hospital with a chief complaint of primary infertility. Her birth and peri-neonatal periods were uneventful, and she experienced menarche with regular menstruation lasting 7 days every 26-31 days. Her medical history included diagnosis of left ovarian endometriosis and she had taken low dose contraceptives. After she married, she had visited another hospital with a complaint of infertility, and she was referred to our hospital for treatment. Physical examination showed normal female physique and external genitalia. Speculum examination revealed a 7 cm blind vaginal canal of normal caliber with two bilateral 2-3 mm pinpoint perforating holes and no visualization of the cervix [Figure 1]. Transvaginal ultrasonography and MRI showed a normal cervix and uterus and 2.5 cm left ovarian endometrioma, but the septum of the vagina was not detected [Figure 2]. The urinary system was normal.

**Figure 3:**
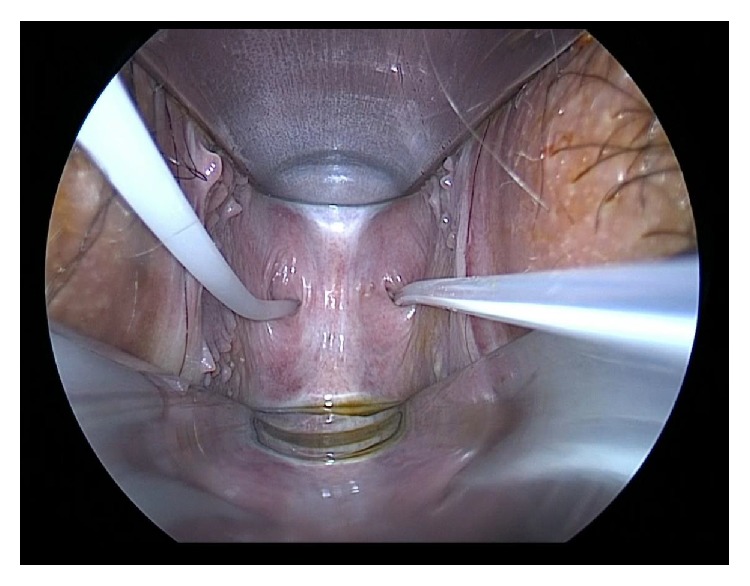
Foley catheters were inserted into the two apertures: 8 French to the left and 14 French to the right. The balloons were then inflated with 3 ml and 8 ml of normal saline to fix each catheter in the proximal vagina.

**Figure 4:**
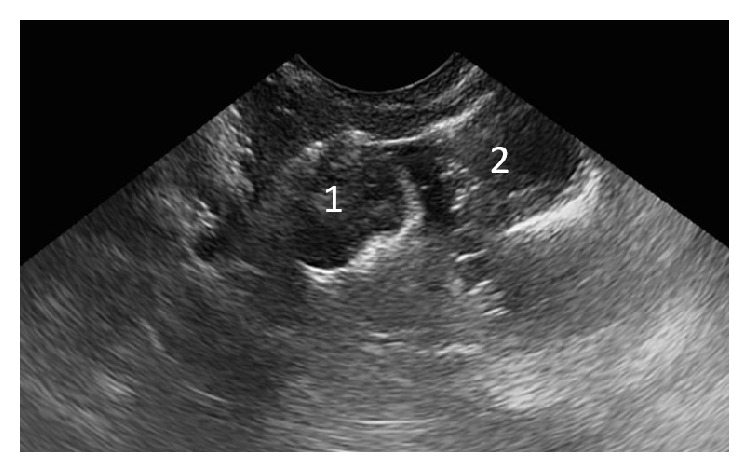
Findings in transabdominal ultrasonography. Each Foley catheter balloon (1) was inflated with normal saline to fix the catheter in the proximal vagina. About 30 ml of saline was injected through the catheters and the proximal vaginal pouch (2) was distended with saline.

**Figure 5:**
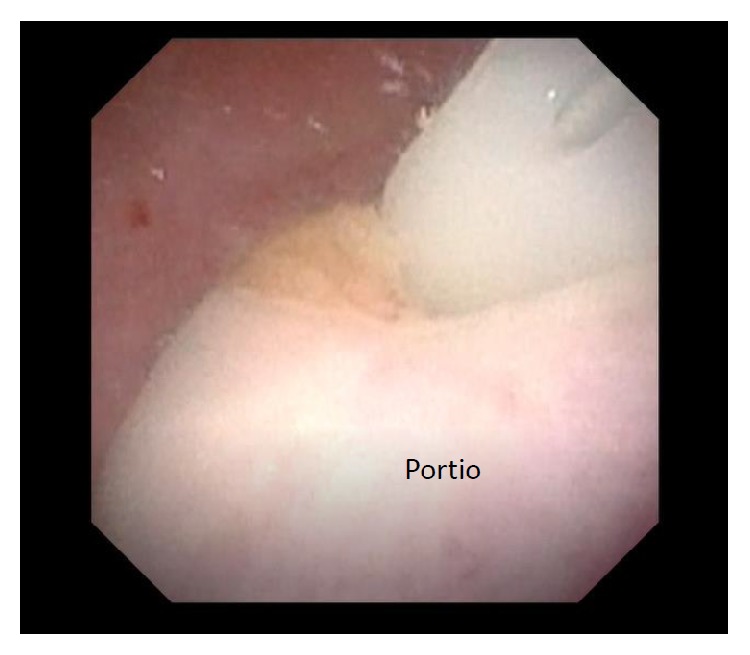
Observation in the vaginal pouch using a flexible endoscope. The vaginal pouch and a completely normal portio were detected.

**Figure 6:**
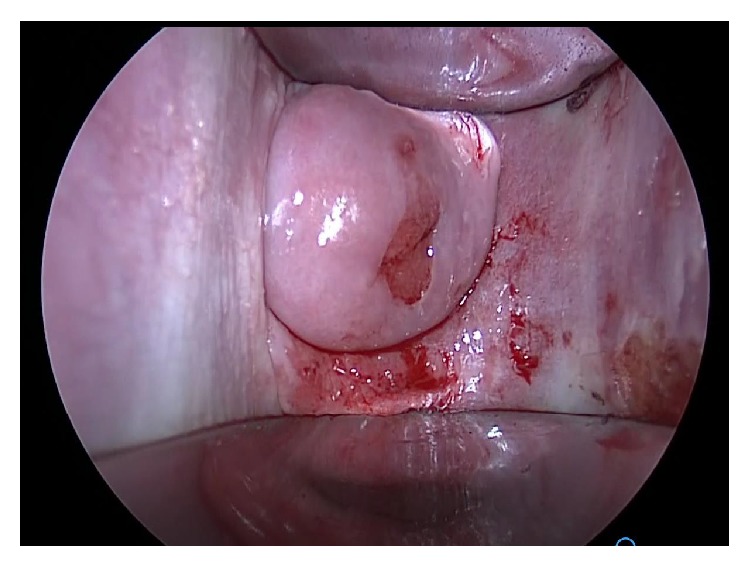
Speculum examination after excision of the septum revealed a normal cervix.

These findings led to suspected perforated TVS of the upper vagina or proximal vagina atresia, and surgical evaluation and treatment were performed under general anesthesia. With the patient placed in a dorsal lithotomy position, Foley catheters were inserted into the two apertures, 8 French to the left and 14 French to the right, and balloons were inflated with 3 ml and 8 ml of normal saline to fix each catheter in the proximal vagina [Figure 3]. About 30 ml of saline was injected through the catheters, and the proximal vaginal pouch distended with saline and the uterine cervix were detected by transabdominal ultrasound [Figure 4]. The Foley catheters were pulled down to prevent water leakage via the perforation of the septum during injection. The findings from saline infusion sonocolpography provided convincing evidence of perforated TVS of the upper vagina, rather than proximal vaginal atresia. To confirm the vaginal pouch and uterine cervix visually, the left Foley catheter was withdrawn and a flexible endoscope was inserted into the vaginal pouch, which revealed a completely normal portio [Figure 5]. Saline infusion sonocolpography revealed that the septum was 3 mm thick and created the proximal vaginal pouch. The septum was excised with cautery and a completely normal cervix was ascertained on speculum examination [Figure 6]. Postoperatively, self-dilation of the vagina was introduced with a dilator made of polyethylene (length 10 cm; diameter 3 cm) for 20 minutes every day for 2 months to prevent agglutination of the excised area. The postoperative course was uneventful.

## 3. Discussion

Absence of the vagina with or without a vaginal blind pouch is a well-known anomaly that may be due to vaginal aplasia, vaginal atresia, or complete TVS [3]. In vaginal aplasia and atresia, fertility is possible depending on the presence of a functioning uterus by performing vaginal construction. In contrast, in TVS the uterus is usually retained and normal genital function is maintained after treatment of the vaginal anomaly. Therefore, proper diagnosis and treatment for the vaginal anomaly is crucial since these kinds of anomalies are operable and curable.

TVS is a particularly rare anomaly of the female genital tract, and its embryonic origin in the vagina remains controversial. Ruggeri et al. classified different vaginal malformations and connected these classes to embryonic, anatomic and surgical criteria. This classification supported the theory that TVS is a vaginal malformation derived from failure of fusion of the Mullerian ducts [4]. TVS can occur at several depths in the vagina. In a study of 42 patients, Lodi found incidences of 45%, 40% and 14% in the upper, middle, and lower thirds, respectively, with imperforated TVS in 3 (7%) and perforated TVS in 39 (92%) of these patients [5]. As stated above, imperforated obstructive vaginal malformation may cause retrograde menstruation and can be easily diagnosed based on clinical manifestations such as amenorrhea and hematocolpometra. In contrast, perforating TVS may be asymptomatic. Patients may have no complaints and diagnosis may be difficult in cases without vaginal fluid retention owing to incomplete obstruction. Moreover, perforating TVS is usually a ring or circular stenosis with a central hole, but perforating apertures may locate laterally in case of TVS with two holes as described in a previous report [6]. However, the classification of female genital tract congenital anomalies like the ESHRE/ESGE consensus does not demonstrate detailed anatomical features such as the configuration of stenosis, the location of perforation and clarification of the exclusions, which sometimes leads to confusion for the diagnosis [7].

CT, MRI, and conventional ultrasound have certain limitations in diagnosis of genital anomalies, with difficulty in differentiation among atresia, aplasia and TVS in these examinations [8, 9]. In our case, as the septum was perforated with two holes, symptoms such as hematocolpometra were not obvious. MRI is reliable for imaging vaginal anomalies and showed a normal cervix and uterus in our case, but the septum of the vagina was not detected by MRI. Thus, in cases with a lack of fluid accumulation due to incomplete vaginal septum and a thin septum, diagnosis of TVS may also be difficult with MRI.

To resolve these difficulties in diagnosis of TVS, we used saline infusion sonocolpography, which revealed a blind vaginal pouch and enabled diagnosis of perforated TVS. This technique is easy to perform at low cost, and provides important additional information, such as the thickness and location of the septum and the presence or absence of the cervix, which allows differentiation between TVS and congenital absence of the cervix. Consequently, surgical resection of the septum was selected for treatment, and this resulted in successful restoration of the genital tract anatomy.

However, our report has a limitation of the diagnostic ambiguity. Acien and Acien postulated the necessity to analyze the embryology and to examine histologically for the better understanding and proper diagnosis of female genital tract malformations [10]. Besides, Suidan and Azoury introduced the hypothesis that epithelium of the vagina and the transverse septum are of mesonephroid origin and identified as such characteristics of the epithelium that lines them [11]. In our case, a histological examination of the septal tissues could not be performed because we did not think it was necessary for the proper diagnosis at the time of the procedure, and this procedural disadvantage falls short of identifying embryonic origin of TVS in our case.

In conclusion, we experienced a patient with perforated TVS in whom successful diagnosis and treatment were achieved using the new technique of saline infusion sonocolpography. This method may be useful for diagnosis and treatment of TVS, for differentiation of this condition from congenital vaginal anomaly, and for planning of associated treatment.
